# Plasma EBV DNA as a prognostic factor in EBV associated gastric cancer: a multicenter, prospective study (EBV PRESAGE study)

**DOI:** 10.3389/fonc.2023.1276138

**Published:** 2023-10-24

**Authors:** Andrea Alberti, Gertraud Stocker, Florian Lordick, Ulrich T. Hacker, Benjamin Kobitzsch, Ivonne Haffner, Gian Luca Baiocchi, Manuel Zamparini, Guido A. M. Tiberio, Carla Baronchelli, Arnaldo Caruso, Paolo Bossi, Alfredo Berruti

**Affiliations:** ^1^ Medical Oncology Unit, Department of Medical and Surgical Specialties, Radiological Sciences, and Public Health, University of Brescia at the Azienda Socio Sanitaria Territoriale (ASST)-Spedali Civili, Brescia, Italy; ^2^ Department of Medicine II (Oncology, Gastroenterology, Hepatology and Pulmonology), University Cancer Center Leipzig (UCCL), University of Leipzig Medical Center, Leipzig, Germany; ^3^ Surgical Unit, Department of Clinical and Experimental Sciences, University of Brescia at the Azienda Socio Sanitaria Territoriale (ASST), Cremona, Italy; ^4^ Surgical Unit, Department of Medical and Surgical Specialties, Radiological Sciences, and Public Health, University of Brescia at the Azienda Socio Sanitaria Territoriale (ASST)-Spedali Civili, Brescia, Italy; ^5^ Pathology Unit, Azienda Socio Sanitaria Territoriale (ASST)-Spedali Civili, Brescia, Italy; ^6^ Microbiology Unit, Department of Molecular and Translational Medicine, University of Brescia-Spedali Civili at the Azienda Socio Sanitaria Territoriale (ASST) – Spedali Civili, Brescia, Italy; ^7^ Medical Oncology and Hematology Unit, Humanitas University, IRCCS Humanitas Research Hospital, Milan, Italy

**Keywords:** gastric and gastroesophageal junction adenocarcinoma, epstein barr virus DNA, prognostic factor, disease-free survival, prospective multicenter study

## Abstract

**Purpose:**

The Cancer Genome Atlas Research Network identified Epstein-Barr-Virus (EBV)-positive gastric cancer as a distinct molecular subtype. The prevalence is 8-9% and the histological examination shows pronounced lymphocytic infiltration, elevated levels of IFN-γ and consequently overexpression of PD-L1. The role of plasma EBV DNA load as a prognostic factor in patients with this cancer subtype is still to be defined.

**Methods and analysis:**

The present multicenter prospective observational study “EBV PRESAGE”, involving German and Italian cancer centers, aims to evaluate the prognostic role of plasma EBV DNA in EBV-related gastric cancer (GC). The objective is to study the association between plasma EBV DNA load at different consecutive time points and the patient’s prognosis. Every patient with a new diagnosis of gastric cancer (including gastroesophageal junction adenocarcinoma) will be screened for Epstein-Barr encoded small Region (EBER) on tissue biopsies using *in situ* hybridization (ISH). If EBER ISH is positive, blood analysis for plasma EBV DNA will be conducted. The plasma EBV quantitative analysis will be centralized, and extraction, detection, and quantification of EBV DNA in plasma samples will be performed using real-time PCR.

**Discussion:**

We hypothesized that plasma EBV DNA represents a non-invasive tool for monitoring EBV-related GC and might be valuable as a prognostic marker.

## Introduction

Gastric cancer (GC) is the 4th most common cancer worldwide and the second leading cause of cancer-related death with 700,000 deaths reported annually (1). The 5-year survival rate in stages I-II is 92-96%, however, resectable disease is detected in only 20-30%, and the recurrence rate after resection is high (1). For advanced disease, the reported 1-year survival is approximately 30% (2). The Cancer Genome Atlas Research Network (TCGA) has identified four distinct molecular subtypes of GC: EBV-positive, microsatellite instable, genomically stable and chromosomal instability; 9% of tumors were identified as EBV-positive (3). A meta-analysis involving 9,738 patients from 48 studies revealed an EBV-positive rate of 8.8% in GC (4). Another pooled analysis included 4,599 GC patients from 13 studies and found an 8.2% EBV positivity rate (5). The EBV subtype is more frequent in males and is usually localized in the gastric fundus or body. The histological examination shows pronounced lymphocytic infiltration, elevated levels of IFN-γ and consequently overexpression of PD-L1 (3, 6). EBV positive GCs have a higher prevalence of DNA hypermethylation than any other GC subtype. In particular, they display CDKN2A (p16INK4A) promoter hypermethylation but lack the MLH1 hypermethylation characteristic of MSI-associated CpG island methylator phenotype (3, 6).

Studies on the prognostic significance of EBV in GC are limited by the small number of EBV-positive tumors; results have been inconsistent, with some reporting a significant survival advantage for EBV-positive tumors (7–12), while others report a non-significant increased risk of death (13–15). Liu et al. conducted a meta-analysis on this aspect and concluded that patients with EBV-positive GC have a significantly better survival than those with EBV-negative cancers (HR 0.67, CI 055-0.79) (16). The pooled analysis by Camargo et al. came to consistent conclusions (HR 0.72; 95% CI 0.61-0.86), and although the stage was inversely associated with EBV positivity, the prognostic impact is preserved in early GC as in advanced GC (5).

Serum tumor markers, like carcinoembryonic antigen and cancer antigen 19-9, are of limited utility for the management of GC. They may be elevated in some patients and a drop in blood levels may correlate with response to preoperative therapy (17). However, clinical decisions are rarely taken based on tumor marker changes alone (17). Likewise, in many (but not all) studies, preoperative elevation in serum tumor markers are an independent indicator of adverse prognosis (17–19). While tumor markers should not be used to exclude a patient from surgical approach. Accordingly, recommendations for preoperative evaluation and staging of GC from the NCCN and ESMO do not include an assay of any tumor marker (20, 21).

Plasma EBV-DNA load has been well documented as an effective marker for distinguishing infectious mononucleosis from the presence of EBV in benign B lymphocytes, as well as for diagnostic screening and monitoring of EBV-associated diseases such as lymphoma, post-transplantation lymph proliferative disease and nasopharyngeal carcinoma (22–25). Especially in nasopharyngeal cancer patients, the quantification of plasma EBV DNA can be used to monitor recurrence and predict prognosis (13). Little has been done to evaluate the role of plasma EBV-DNA load in the diagnosis and monitoring of GC. Shoda et al. evaluated the clinical utility of plasma EBV-DNA in EBV-associated GC and found that the sensitivity and specificity of detection were 71.4% and 97.1%, respectively (26). An Asian prospective cohort study from a single cancer center in China identified 140/2760 (5.1%) EBER-positive GC. This study confirmed that EBV-associated GC had better overall survival and suggested that elevated dynamic detection of plasma EBV-DNA load offered an easily accessible biomarker to monitor EBV-associated GC (27). However, there are few data on the role of plasma EBV DNA load as a prognostic or predictive biomarker for EBV-associated GC in a non-Asian population.

Herein, we describe the design and methodology of the EBV PRESAGE study, an international, prospective observational trial, evaluating the prognostic role of plasma EBV DNA in EBV-related GC.

## Materials and analysis

### Hypothesis and objectives

The primary hypothesis is to identify the association between pre-treatment plasma EBV DNA load and the patient’s prognosis in a series of consecutive, prospective early EBV-related GC (stage Ib-IIIc). Secondary objective is to evaluate the role of EBV DNA load after surgery as a prognostic factor for disease-free survival. Moreover, descriptive objectives are to evaluate the possibility to test circulating plasmatic EBV DNA at baseline and at consecutive time points during follow-up and during systemic treatments in advanced (stage IV) EBV-related GC.

### Eligibility criteria

The study population includes female and male patients, age ≥ 18 years, diagnosed with early (stage Ib-IIIc) or advanced (stage IV) GC (including gastroesophageal junction adenocarcinoma). The study enrolls patients who are going to start a preoperative or perioperative or a 1st – 2nd line systemic therapy, as well as those who are only candidates for surgical treatment.

### Study procedures

**Figure 1:**
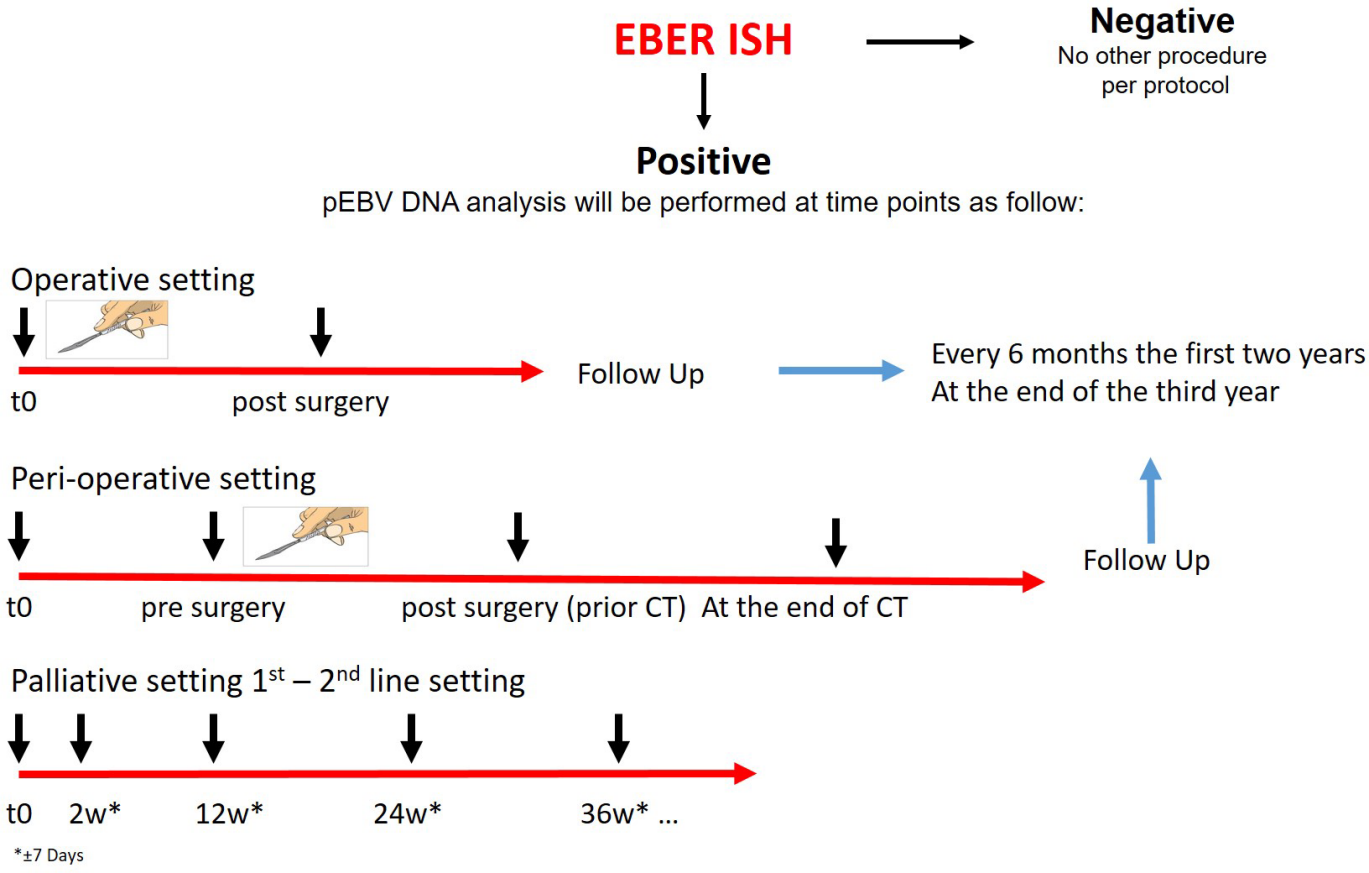
Study design. CT chemotherapy, EBV Epstein Barr virus, EBER Epstein-Barr Encoding Region, ISH in situ hybridization. Patients with GC will be screened with EBER ISH. If EBER ISH is positive, blood analysis for plasma EBV DNA will be conducted. Plasma EBV DNA analysis will be performed according to the setting at different times. During follow up blood samples will be collected every 6 months the first two years and at the end of the third year.

Every patient will be screened for EBV by EBER-ISH on tissue biopsy. If EBER ISH is positive, blood analysis for plasma EBV DNA will be conducted. Patients enrolled will be divided according to the type of treatment proposed in operative, perioperative, and palliative categories. Those undergoing surgical treatment with or without any peri-operative treatment will be evaluated for disease free survival analysis. The blood samples will be collected in these categories before the start of any treatment. Moreover, plasma will be collected after surgery and it will be evaluated as a prognostic factor for disease-free survival. In order to describe the changes of plasma EBV DNA levels and correlate with the radiological results, blood samples will be collected during follow-up, approximately every 6 months for the first two years and at the end of the third year. In the advanced setting, this is an exploratory study to describe the trend of plasma EBV DNA during the first and consecutive lines of therapy. Plasma will be collected at the second week after the start of treatment to evaluate early changes and then every 12 weeks to evaluate the clearance rate until progression. **Figure 1** shows the study design.

### EBER ISH and plasma EBV DNA PCR

Archival tumor tissue, obtained for clinical practice, will be used for EBER ISH. The test will be performed locally, at each pathological anatomy department of participant centers. EBER detection will be performed on FFPE sample by an ISH kit according to the manufacturer’s specification.

The plasma EBV quantitative analysis will be centralized at the Laboratory of Microbiology of Spedali Civili of Brescia. Extraction, detection and quantification of EBV DNA in plasma samples will be performed using a completely automatized platform based on RT PCR technology (EBV ELITe MGB^®^ Kit). Targets of RT PCR are region of Epstein-Barr virus nuclear antigen 1 (EBNA 1) gene and human beta-globin gene as an internal control.

### Planned sample size

To assess the prognostic value of plasma EBV DNA load at baseline (pre-treatment) and post-treatment for disease free survival in early EBV-related GC we will use a Cox model. Assuming a log(copies/ml) standard deviation for EBV-DNA of approximately 8.5, an R2 with the other variables (covariates) of 0.2, a baseline 3 years survival of 50% and a HR >= 1.1 for a unit increase in log(EBV-DNA), alpha=5% and power 80%, we would have N=31 patients. Considering a drop-out rate of 10%, 35 patients will be enrolled. The enrollment period is from January 2023 to January 2026; the planned follow-up period is 3 years, so the study duration is 6 years.

During follow-up and in the advanced setting EBV DNA load measured at different time intervals will be described as mean (sd) and median (range), and to represent the trend of plasma EBV DNA in the different time points (descriptive objectives) we will use the raw and percentages variations, testing their difference from 0 through one sample t-test or alternatively the non-parametric sign test, after checking variables distributions. All analyses will be done with the software SAS, SPSS, and R.

## Discussion

It is known that 5-9% of GC are EBV-related. The EBV subtype has a distinct molecular profile, a favourable survival, and the EBV-association seems to be a predictive biomarker for immunotherapy (3, 5, 16, 27, 28). In an Asian population it was shown that dynamic detection of EBV DNA plasma load offers an easily accessible biomarker to monitor GC (26, 27). However, knowledge about the plasma EBV DNA in gastric cancer is still limited and prospective data from Non-Asian populations are lacking. Therefore, we designed the EBV PRESAGE study to investigate the role of plasma EBV DNA in a non-Asian EBV-related GC population.

We hypothesize that like in nasopharyngeal cancer, also in EBV-related GC, there could be an association between plasma EBV DNA levels and survival. Such a prognostic factor could help stratify treatment according to the expected prognosis. Subsequent EBV DNA assessments could also inform current practice to quantify the response to peri-operative treatment and to stratify according to the risk of recurrence. In the future, this information could be used to personalize peri-operative management and integrate plasma EBV DNA into clinical follow-up. In the metastatic setting, this biomarker could help in evaluating treatment response and anticipate disease progression. In conclusion, we hypothesize that data from this study support the evidence that plasma EBV DNA represents a non-invasive tool for monitoring EBV-related GC.

## Data availability statement

The original contributions presented in the study are included in the article/supplementary material, further inquiries can be directed to the corresponding authors.

## Ethics statement

The institutional review board at each participating site has approved the EBV PRESAGE study protocol and relevant supplementary information. The trial is performed in accordance with ethical principles that have their origin in the Declaration of Helsinki and are consistent with the International Council of Harmonisation/Good Clinical Practice and applicable regulatory requirements. Written informed consent will be obtained from each trial participant. The results of this study will be published in international, peer-reviewed journals, and presented at national and international conferences.

## Author contributions

AA: Writing – original draft. GS: Writing – review & editing. FL: Writing – review & editing. UH: Writing – review & editing. BK: Writing – review & editing. IH: Writing – review & editing. GB: Writing – review & editing. MZ: Writing – review & editing. GT: Writing – review & editing. CB: Writing – review & editing. AC: Writing – review & editing. PB: Writing – review & editing. AB: Writing – review & editing.
